# Supplementary material for: Modeling glioblastoma heterogeneity as a dynamic network of cell states
Source: Mol Syst Biol. 2021 Sep 16;17(9):e10105. doi: 10.15252/msb.202010105 (PMC8444284; doi:10.15252/msb.202010105)
Supplement: Supplementary file 6 — Source Data for Figure 5 [file MSB-17-e10105-s004.zip › Figure5A_sourcedata/GSEA_3071/nosigdb_stateV.GseaPreranked.1622795159954/LARSSON_STATE_4.html]

Details for gene set LARSSON\_STATE\_4[GSEA]

|  || Dataset | state43071 |
| Phenotype | NoPhenotypeAvailable |
| Upregulated in class | na\_pos |
| GeneSet | LARSSON\_STATE\_4 |
| Enrichment Score (ES) | 0.8072219 |
| Normalized Enrichment Score (NES) | 4.3584633 |
| Nominal p-value | 0.0 |
| FDR q-value | 0.0 |
| FWER p-Value | 0.0 |
Table: GSEA Results Summary

  

Fig 1: Enrichment plot: LARSSON\_STATE\_4      
 Profile of the Running ES Score & Positions of GeneSet Members on the Rank Ordered List

  

| PROBE | GENE SYMBOL | GENE\_TITLE | RANK IN GENE LIST | RANK METRIC SCORE | RUNNING ES | CORE ENRICHMENT || 1 | HIST1H1B |  |  | 0 | 2.500 | 0.0204 | Yes |
| 2 | HIST1H4C |  |  | 1 | 2.442 | 0.0404 | Yes |
| 3 | ATAD2 |  |  | 2 | 2.214 | 0.0585 | Yes |
| 4 | ESCO2 |  |  | 3 | 2.193 | 0.0764 | Yes |
| 5 | HMGB2 |  |  | 5 | 2.011 | 0.0927 | Yes |
| 6 | NUSAP1 |  |  | 6 | 2.001 | 0.1090 | Yes |
| 7 | HIST1H1C |  |  | 7 | 1.976 | 0.1252 | Yes |
| 8 | PCNA |  |  | 10 | 1.908 | 0.1404 | Yes |
| 9 | TOP2A |  |  | 14 | 1.842 | 0.1549 | Yes |
| 10 | HIST1H1E |  |  | 15 | 1.830 | 0.1698 | Yes |
| 11 | MCM4 |  |  | 17 | 1.815 | 0.1845 | Yes |
| 12 | DHFR |  |  | 18 | 1.795 | 0.1991 | Yes |
| 13 | HIST1H1D |  |  | 19 | 1.786 | 0.2137 | Yes |
| 14 | NCAPG |  |  | 21 | 1.760 | 0.2279 | Yes |
| 15 | RAD51AP1 |  |  | 22 | 1.757 | 0.2423 | Yes |
| 16 | H2AFX |  |  | 26 | 1.725 | 0.2558 | Yes |
| 17 | PCLAF |  |  | 27 | 1.720 | 0.2699 | Yes |
| 18 | CENPF |  |  | 28 | 1.705 | 0.2838 | Yes |
| 19 | TYMS |  |  | 30 | 1.683 | 0.2973 | Yes |
| 20 | RRM2 |  |  | 34 | 1.660 | 0.3103 | Yes |
| 21 | MYBL2 |  |  | 35 | 1.651 | 0.3238 | Yes |
| 22 | CENPU |  |  | 36 | 1.645 | 0.3373 | Yes |
| 23 | SGO1 |  |  | 40 | 1.614 | 0.3499 | Yes |
| 24 | E2F1 |  |  | 44 | 1.602 | 0.3624 | Yes |
| 25 | MKI67 |  |  | 45 | 1.598 | 0.3755 | Yes |
| 26 | BRCA1 |  |  | 46 | 1.592 | 0.3885 | Yes |
| 27 | MCM3 |  |  | 49 | 1.586 | 0.4011 | Yes |
| 28 | KIF15 |  |  | 52 | 1.568 | 0.4135 | Yes |
| 29 | PBK |  |  | 54 | 1.536 | 0.4258 | Yes |
| 30 | EZH2 |  |  | 55 | 1.534 | 0.4384 | Yes |
| 31 | GINS2 |  |  | 57 | 1.528 | 0.4507 | Yes |
| 32 | GTSE1 |  |  | 66 | 1.491 | 0.4613 | Yes |
| 33 | CDK1 |  |  | 69 | 1.488 | 0.4731 | Yes |
| 34 | SMC2 |  |  | 73 | 1.466 | 0.4845 | Yes |
| 35 | CHAF1A |  |  | 77 | 1.441 | 0.4957 | Yes |
| 36 | UBE2T |  |  | 79 | 1.432 | 0.5072 | Yes |
| 37 | FBXO5 |  |  | 82 | 1.422 | 0.5185 | Yes |
| 38 | CLSPN |  |  | 84 | 1.417 | 0.5298 | Yes |
| 39 | MCM7 |  |  | 88 | 1.400 | 0.5407 | Yes |
| 40 | MCM10 |  |  | 92 | 1.389 | 0.5515 | Yes |
| 41 | DEK |  |  | 94 | 1.384 | 0.5626 | Yes |
| 42 | RRM1 |  |  | 99 | 1.364 | 0.5730 | Yes |
| 43 | CENPM |  |  | 114 | 1.337 | 0.5812 | Yes |
| 44 | SMC4 |  |  | 127 | 1.320 | 0.5897 | Yes |
| 45 | TMSB15A |  |  | 135 | 1.306 | 0.5990 | Yes |
| 46 | GMNN |  |  | 146 | 1.280 | 0.6075 | Yes |
| 47 | TMPO |  |  | 161 | 1.243 | 0.6150 | Yes |
| 48 | CENPK |  |  | 162 | 1.241 | 0.6252 | Yes |
| 49 | CDKN3 |  |  | 163 | 1.239 | 0.6353 | Yes |
| 50 | LMNB1 |  |  | 165 | 1.238 | 0.6452 | Yes |
| 51 | DBF4 |  |  | 189 | 1.197 | 0.6506 | Yes |
| 52 | KIF4A |  |  | 191 | 1.193 | 0.6601 | Yes |
| 53 | FANCI |  |  | 196 | 1.179 | 0.6690 | Yes |
| 54 | ZWINT |  |  | 204 | 1.166 | 0.6772 | Yes |
| 55 | UBE2C |  |  | 214 | 1.154 | 0.6849 | Yes |
| 56 | CDC45 |  |  | 228 | 1.138 | 0.6917 | Yes |
| 57 | ASF1B |  |  | 237 | 1.130 | 0.6994 | Yes |
| 58 | MCM5 |  |  | 243 | 1.127 | 0.7076 | Yes |
| 59 | SGO2 |  |  | 244 | 1.126 | 0.7168 | Yes |
| 60 | TPX2 |  |  | 248 | 1.122 | 0.7254 | Yes |
| 61 | STMN1 |  |  | 307 | 1.062 | 0.7229 | Yes |
| 62 | DUT |  |  | 314 | 1.050 | 0.7304 | Yes |
| 63 | RFC2 |  |  | 337 | 1.025 | 0.7345 | Yes |
| 64 | PIMREG |  |  | 339 | 1.025 | 0.7427 | Yes |
| 65 | PHF19 |  |  | 378 | 0.971 | 0.7433 | Yes |
| 66 | UHRF1 |  |  | 390 | 0.958 | 0.7490 | Yes |
| 67 | RPA3 |  |  | 395 | 0.953 | 0.7561 | Yes |
| 68 | NUDT1 |  |  | 417 | 0.936 | 0.7597 | Yes |
| 69 | MAD2L1 |  |  | 421 | 0.932 | 0.7667 | Yes |
| 70 | KPNA2 |  |  | 435 | 0.919 | 0.7717 | Yes |
| 71 | CENPH |  |  | 448 | 0.908 | 0.7768 | Yes |
| 72 | ORC6 |  |  | 449 | 0.908 | 0.7843 | Yes |
| 73 | HMGN2 |  |  | 466 | 0.900 | 0.7885 | Yes |
| 74 | LSM3 |  |  | 468 | 0.892 | 0.7956 | Yes |
| 75 | MND1 |  |  | 480 | 0.885 | 0.8007 | Yes |
| 76 | UBE2S |  |  | 514 | 0.854 | 0.8014 | Yes |
| 77 | BIRC5 |  |  | 541 | 0.842 | 0.8033 | Yes |
| 78 | HMGB1 |  |  | 583 | 0.810 | 0.8020 | Yes |
| 79 | TK1 |  |  | 591 | 0.806 | 0.8072 | Yes |
| 80 | SIVA1 |  |  | 646 | 0.774 | 0.8032 | No |
| 81 | HNRNPD |  |  | 659 | 0.769 | 0.8071 | No |
| 82 | DTYMK |  |  | 800 | 0.700 | 0.7859 | No |
| 83 | TUBB |  |  | 836 | 0.685 | 0.7848 | No |
| 84 | ALYREF |  |  | 847 | 0.680 | 0.7884 | No |
| 85 | H2AFZ |  |  | 851 | 0.677 | 0.7934 | No |
| 86 | H2AFV |  |  | 933 | 0.647 | 0.7831 | No |
| 87 | PTTG1 |  |  | 936 | 0.647 | 0.7880 | No |
| 88 | CENPN |  |  | 953 | 0.638 | 0.7901 | No |
| 89 | CKS2 |  |  | 966 | 0.634 | 0.7930 | No |
| 90 | RANBP1 |  |  | 999 | 0.620 | 0.7919 | No |
| 91 | LSM4 |  |  | 1003 | 0.619 | 0.7964 | No |
| 92 | TUBA1B |  |  | 1012 | 0.617 | 0.7999 | No |
| 93 | NUCKS1 |  |  | 1178 | 0.570 | 0.7728 | No |
| 94 | ANP32B |  |  | 1187 | 0.566 | 0.7759 | No |
| 95 | CKS1B |  |  | 1502 | 0.479 | 0.7194 | No |
| 96 | HIST1H1A |  |  | 1533 | 0.473 | 0.7175 | No |
| 97 | LSM5 |  |  | 1982 | 0.391 | 0.6345 | No |
| 98 | CCNB1 |  |  | 2480 | 0.325 | 0.5416 | No |
Table: GSEA details [plain text format]

  

Fig 2: LARSSON\_STATE\_4: Random ES distribution      
 Gene set null distribution of ES for **LARSSON\_STATE\_4**
